# Fast and Efficient Piezo/Photocatalytic Removal of Methyl Orange Using SbSI Nanowires

**DOI:** 10.3390/ma13214803

**Published:** 2020-10-28

**Authors:** Krystian Mistewicz, Mirosława Kępińska, Marian Nowak, Agnieszka Sasiela, Maciej Zubko, Danuta Stróż

**Affiliations:** 1 Institute of Physics–Center for Science and Education, Silesian University of Technology, 40-019 Katowice, Poland; miroslawa.kepinska@polsl.pl (M.K.); marian.nowak@polsl.pl (M.N.); agnisas480@student.polsl.pl (A.S.); 2 Institute of Materials Engineering, Faculty of Science and Technology, University of Silesia, 41-500 Chorzów, Poland; maciej.zubko@us.edu.pl (M.Z.); danuta.stroz@us.edu.pl (D.S.); 3 Department of Physics, Faculty of Science, University of Hradec Králové, 500 03 Hradec Králové, Czech Republic

**Keywords:** ultrasound-assisted piezocatalysis, antimony sulfoiodide (SbSI), nanowires, methyl orange, water purification

## Abstract

Piezocatalysis is a novel method that can be applied for degradation of organic pollutants in wastewater. In this paper, ferroelectric nanowires of antimony sulfoiodide (SbSI) have been fabricated using a sonochemical method. Methyl orange (MO) was chosen as a typical pollutant, as it is widely used as a dye in industry. An aqueous solution of MO at a concentration of 30 mg/L containing SbSI nanowires (6 g/L) was subjected to ultrasonic vibration. High degradation efficiency of 99.5% was achieved after an extremely short period of ultrasonic irradiation (40 s). The large reaction rate constant of 0.126(8) s^−1^ was determined for piezocatalytic MO decomposition. This rate constant is two orders of magnitude larger than values of reaction rate constants reported in the literature for the most efficient piezocatalysts. These promising experimental results have proved a great potential of SbSI nanowires for their application in environmental purification and renewable energy conversion.

## 1. Introduction

The pollution of water is recognized as one of the major global problems that have to be solved in the near future. Textile, printing, pharmaceutical, and food industries require use of many various dyes, including methyl orange (MO). An application of these organic dyes may lead to water contamination, which results in serious health and environmental problems. A removal of the toxic organic compounds from wastewater can be realized using different techniques, such as advanced oxidation process [1]. However, traditional oxidation methods encounter major problems like the high operational cost of the popular ozonation technique and production of ion-based sludge as an undesirable result of Fenton oxidation.

Piezoelectrically induced catalysis, known as piezocatalysis [2,3], is a new alternative method for contaminants degradation. In this process, a mechanical energy (e.g., ultrasonic vibration) is converted into chemical energy or an external mechanical-force-induced piezopotential [4,5] promotes photocatalytic activity of piezoelectric material [6,7]. Over the last several years, a lot of different piezoelectric materials have been proposed for application in ultrasound-assisted piezocatalysis, including BaTiO_3_ nanostructures [8,9,10,11], SrTiO_3_ nanocrystals [12], BiFeO_3_ micro-sheets [13], Bi_2_WO_6_ nanosheets [14], MoS_2_ nanoflowers [15], Na_0.5_K_0.5_NbO_3_ [16], NiO nanoparticles [17], Pb(Zr,Ti)O_3_ (PZT) nanowires [18], ZnO nanostructures [19,20], ZnS nanosheets [21], and ZnSnO_3_ nanoparticles [22]. It has been well documented that the piezoelectric potential of one- or two-dimension piezoelectric materials is larger than that of particles due to higher deformation ability [9]. However, one of the most important issues for blocking the commercial applications of the aforementioned piezocatalysts is the long ultrasonic vibration required for achieving a sufficient level of pollutant degradation. For example, Jin et al. [8] have recently prepared BaTiO_3_ nanowires by the sol-gel-based electrospinning technique and used this ferroelectric material for a degradation of methyl orange solution under ultrasonic vibration. They have found that the piezocatalytic activity of BaTiO_3_ nanowires is highly sensitive to its microstructure, including phase structure, crystallite size, specific surface area and nanowire diameter [8]. The highest piezocatalytic efficiency of 95% has been achieved after 160 min of reaction for nanowires with the least diameters.

Antimony sulfoiodide (SbSI) is ferroelectric semiconductor with Curie temperature of 291(2) K [23]. SbSI possesses excellent piezoelectric properties confirmed by a large piezoelectric modulus (1 nC/N [24]), huge electromechanical coupling coefficient (0.9 [25]), and high electrostrictive constant (4.6(1)·10^−13^ m^2^/V^2^ [26]). Therefore, nanowires of SbSI have been successively applied in piezoelectric generators for mechanical energy harvesting [27,28,29]. Moreover, SbSI nanowires have been found as a suitable material for photovoltaic devices [23,30] and gas sensors [31,32,33].

The nanostructures of SbSI have been recently recognized as excellent visible light photocatalysts [34,35,36] with efficient generation of singlet oxygen. In 2018, Wang et al. [34] presented that 99% of methyl orange in aqueous solution with concentration of 30 mg/L was degraded in an extremely short time of 10 s using small amounts of SbSI nanocrystals (1 g/L). Two years earlier, Muthusamy and Bhattacharyya [35] proposed novel solution-based synthesis of self-assembled micron-sized sea “urchin” shaped SbSI with a high density of 1D micron-sized rods. Prepared material displayed a powerful photodegradation of MO in aqueous solutions under visible light irradiation. The highly efficient degradation activity on the organic pollutant was accounted by the formation of lower dangling bond on the surface of SbSI microrods as well as by the large static dielectric constant generated by the cross band hybridization between the ns^2^ (Sb^3+^) cation and iodine ion [35]. Tasviri and Sajadi-Hezave [36] demonstrated a strong photocatalytic activity of SbSI nanowires and carbon nanotubes encapsulated with SbSI toward decomposition of Acid Blue 92 (monoazo dye).

In this paper, for the first time, SbSI nanowires have been discovered to exhibit a piezocatalytic effect that can be successively applied for destroying of MO dye in aqueous solutions. The kinetics of piezo- and photocatalysis has been studied. The reaction rate constants and degradation efficiencies were determined and compared to these parameter of other nanomaterials known in the literature as good piezo- and photocatalysts.

## 2. Materials and Methods

### 2.1. Synthesis of SbSI Nanowires

Nanowires of SbSI were fabricated as the products of sonochemical synthesis similarly to reports provided in [23,30,37]. High purity ethanol with volume of 5 mL, 226 mg of sulfur, 855 mg of antimony, and 970 mg of iodine were inserted into a small plastic vessel. All these reagents were delivered by Avantor Performance Materials Poland company (Gliwice, Poland). The vessel with the suspension of the reagents was exposed for 120 min to high power ultrasounds with frequency of 20 kHz generated by VCX-750 reactor (Sonics & Materials Inc., Newtown, CT, USA). The reaction was carried out at a temperature of 323 K.

### 2.2. Additional Purification Procedure

A special purification process was accomplished to remove probable intermediates that could be present in the fabricated SbSI ethanogel. This procedure was similar to this described in [38]. After sonochemical synthesis was finished, a small amount of SbSI gel of 40 mg was inserted into the MPW M-Diagnostic centrifuge (MPW Med. Instruments, Warsaw, Poland) and rotated at 3200 rpm for 6 min in order to separate the nanowires from the liquid. Then, the extracted liquid phase was withdrawn and the ethanol was introduced into the vessel. Subsequently, SbSI nanowires in ethanol were dispersed ultrasonically for 8 min. The procedure, described above, was repeated 12 times. At the final stage, the SbSI gel was desiccated in the chamber for ten hours at a temperature of 313 K in order to remove the ethanol.

### 2.3. Characterization of the Catalysts

The high-resolution transmission electron microscopy (HRTEM) investigations were carried out on the JEOL JEM 3010 microscope (JEOL USA Inc., Peabody, MA, USA) by applying accelerating voltage of 300 kV. The suspension of SbSI nanowires in isopropanol was irradiated ultrasonically for 30 min. Then, a standard copper grid with a porous amorphous carbon film was coated with prepared suspension of SbSI nanowires. Finally, the sample was dried to evaporate the isopropanol.

Phenom Pro X scanning electron microscope (SEM) (Thermo Fisher Scientific, Waltham, MA, USA) was applied to examine the surface topography of the SbSI nanowires. The concentrations of the chemical elements were quantified using an energy-dispersive X-ray spectroscopy (EDS) detector (Thermo Fisher Scientific, Waltham, MA, USA) and ProSuite Element Identification software (version 1.0, Thermo Fisher Scientific, Waltham, MA, USA).

### 2.4. Piezo- and Photocatalysis Experiments

Methyl orange (C_14_H_14_N_3_NaSO_3_) was chosen as a model compound, a typical pollutant of anionic azo dye. It was obtained from Sigma-Aldrich, Inc. (Saint Louis, MO, USA). For a standard degradation experiment, 90 mg of SbSI nanowires were dispersed in 15 mL of 30 mg/L MO aqueous solution. Nanoparticles of barium titanate (BaTiO_3_) with average size of 100 nm were purchased from Sigma-Aldrich (Saint Louis, MO, USA) and used as a reference catalysts. Before the application of ultrasonic vibration, the SbSI-MO dispersion was agitated in the dark for 20 min to achieve an adsorption-desorption equilibrium between the dye solution and the piezocatalyst. The ultrasonic vibration was performed using two different reactors, i.e., the ultrasonic processor VCX-750 (Sonics & Materials Inc., Newtown, CT, USA) with frequency of 20 kHz and maximum power of 750 W, and the Sonic-6 (Polsonic) reactor with working frequency of 40 kHz and maximum power of 480 W. The small volumes of liquid were withdrawn from the SbSI-MO suspension at certain time intervals. Then, the supernatant liquid was separated from the piezocatalyst using HPLC syringe filters equipped with a nylon membrane with average pore size of 0.2 μm. An optical transmittance of the MO aqueous solutions during the degradation experiment was registered with PC2000 spectrophotometer, DH2000-FHS lamp, and the OOI-Base software (version 1.5, Ocean Optics, Inc., Dunedin, FL, USA). This apparatus was bought from Ocean Optics Inc. (Dunedin, FL, USA). In order to avoid the influence of photocatalysis, the piezocatalytic degradation experiments were carried out in the dark. The catalytic properties of SbSI nanowires were also investigated under light illumination. Additional photocatalytic experiments were accomplished using ultraviolet (UV) lamp (Sineo, Shanghai, China) with power of 300 W.

The optical transmission characteristics of MO solutions were measured several times to confirm their repeatability and stability. Data, presented in the absorbance plots, represent the averaged spectral characteristics. The uncertainties of MO concentrations were calculated taking into account the standard deviation of multiple measurements, the spectral resolution of the PC2000 spectrophotometer (Ocean Optics, Inc., Dunedin, FL, USA) and the experimental error of determination of MO initial concentration. The evaluated uncertainties were presented as error bars in appropriate graphs.

## 3. Results and Discussion

### 3.1. Material Characterization

**Figure 1:**
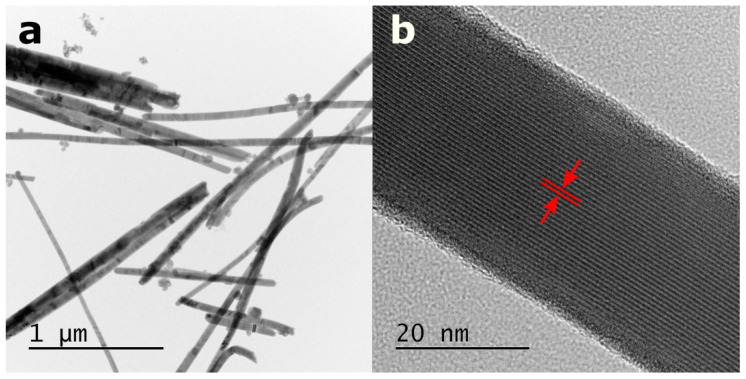
(**a**) TEM micrograph of fabricated SbSI nanowires and (**b**) HRTEM image of the selected nanowire. Marked in red interplanar spacing d = 0.6527(18) nm is consistent with the interplanar spacing of (110) planes reported in the literature for SbSI [39].

**Figure 2:**
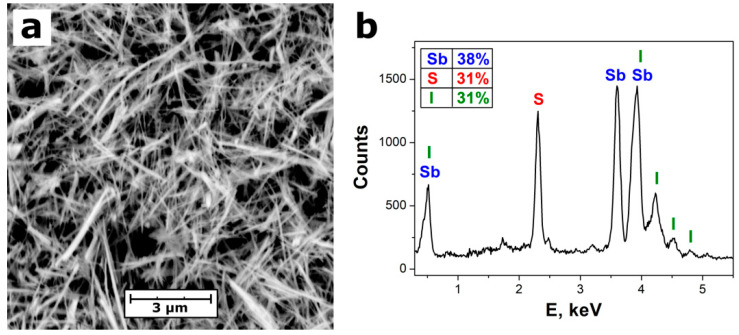
(**a**) Representative SEM micrograph and (**b**) EDS spectrum of SbSI nanowires. Inset in the graph presents the table with determined atomic concentrations of the chemical elements.

Figure 1 and Figure 2a depict the one-dimensional structure of SbSI nanowires confirmed with transmission electron microscopy (TEM) and scanning electron microscopy (SEM), respectively. It was found that the nanowire length is of micrometer scale, while diameters of the majority (88%) of the studied nanowires vary from 40 nm to 100 nm. The evident (110) lattice fringes, parallel to the nanowire axis, were identified in the HRTEM image of an individual SbSI nanowire (Figure 1b). The interplanar spacing of 0.6527(18) nm was determined. Taking into account the experimental uncertainty, the estimated value equals to interplanar spacing d = 0.65244 nm of (110) planes reported in the literature for crystalline structure of SbSI [39]. The crystalline core of SbSI nanowire is covered with an amorphous shell with thickness of a few nanometers (Figure 1b). This feature was frequently documented in other papers on sonochemically prepared SbSI nanowires [30,40] as well as nanocrystals fabricated via ball-milling of bulk SbSI [34].

A representative energy-dispersive X-ray spectroscopy (EDS) spectrum of SbSI nanowires is given in Figure 2b. It consists of sharp peaks. They were assigned exclusively to chemical elements that are expected to be present in SbSI. The atomic concentrations of 38%, 31%, and 31% were quantified for antimony, sulfur, and iodine, respectively. The obtained results are close to the theoretical chemical composition of SbSI, apart from a slight excess amount of antimony. A similar effect was reported in the literature in the case of EDS [31,34] and X-ray photoelectron spectroscopy (XPS) [30] studies of SbSI nanostructures. It can be attributed to the fact that SbSI nanowire is surrounded by very thin fuzzy shell (Figure 1b), which was confirmed by HRTEM survey (Figure 1b). The chemical composition of the amorphous shell may be distinct from the concentrations of the elements in a crystalline core of the nanowire [30,34,40].

### 3.2. Piezo- and Photocatalytic Performance of SbSI Nanowires

**Figure 3:**
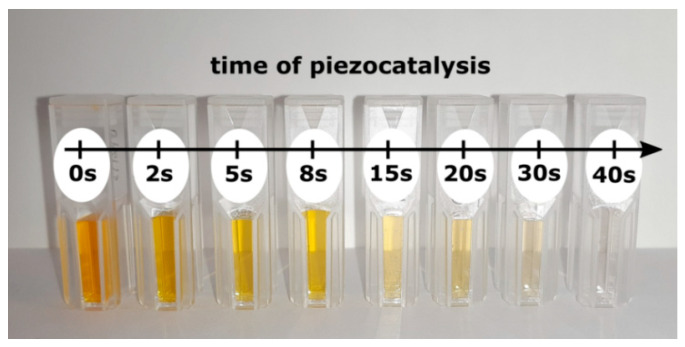
Photograph of the methyl orange (MO) aqueous solutions samples after different times of ultrasonic irradiation. Piezocatalysis was performed using VCX-750 ultrasonic reactor (Sonics & Materials Inc., Newtown, CT, USA) with ultrasound frequency f = 20 kHz and power P = 750 W.

Figure 3 presents an influence of piezocatalysis time on the color of the MO aqueous solutions. It can be clearly observed with the naked eye, that the increase of the time of ultrasonic vibration leads to fading of the tone of the MO solution.

**Figure 4:**
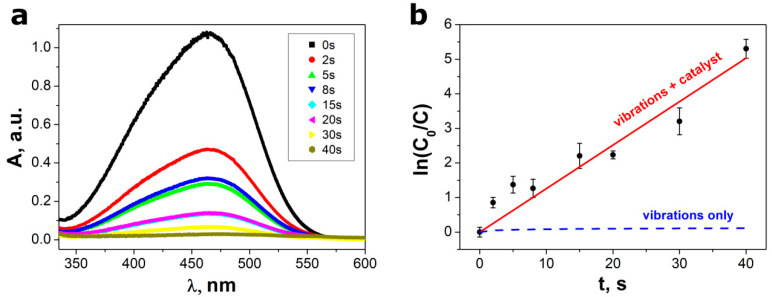
(**a**) An influence of piezocatalysis time on UV–vis absorption spectra of the MO aqueous solutions; (**b**) plots of ln(C_0_/C) versus the ultrasonic vibration time. Red line in graph (**b**) represents the best fitted dependence described by Equation (1). Blue dashed line in graph (**b**) shows data registered in control experiments conducted under ultrasonic vibration without SbSI piezocatalyst. Piezocatalysis was performed using VCX-750 (Sonics & Materials, Inc.) ultrasonic reactor with ultrasound frequency f = 20 kHz and power P = 750 W.

The values of the absorbance were calculated from the optical transmittance using a well-known relationship [41]. The UV–visible spectrum of MO dissolved in distilled water exhibits two absorption maxima, i.e., at 270 nm and 465 nm [42]. The second mentioned peak was used to monitor the effect of the piezocatalysis on the degradation of MO (Figure 4a). One can see that the height of the absorption peak (at λ = 465 nm) in the UV–vis absorption spectra of MO solution decreases gradually with the increase of time of ultrasonic vibration. The complete degradation of MO by SbSI nanowires is achieved after a short ultrasonic irradiation time of 40 s.

The standard Lambert–Beer law calibration curve [43] for methyl orange was used to estimate the MO concentration. Figure 4b presents the relative concentration ratio (C/C_0_) of methyl orange as a function of ultrasonic vibration time, where C_0_ represents the maximum absorption peak of MO at t = 0 s. The reaction kinetics has been found to follow pseudo-first-order law. Therefore, it can be described using following relation [8,9,10,18]:

(1) $$C=C_{0}\exp\left(-k\cdot t\right)$$

where k means the reaction rate constant. The value k_1_ = 0.126(8) s^−1^ was determined by linear fitting in the plot of ln(C_0_/C) versus t, as depicted in Figure 4b. It should be underlined that there was no noticeable influence of the ultrasonic vibrations on concentration of MO dissolved in water without the catalyst (Figure 4b). It is in agreement with numerous papers [8,9,10,11,14,16,18,19,20] reporting that exclusive application of ultrasonic irradiation does not have an impact on the stability of methyl orange in aqueous solutions.

**Figure 5:**
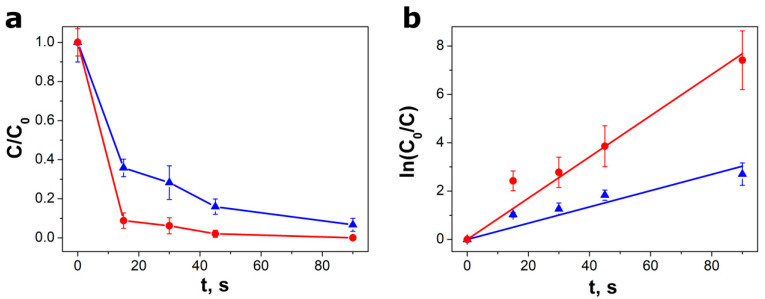
Plots of (**a**) methyl orange relative concentrations C/C_0_ and (**b**) ln(C_0_/C) versus vibration time for two different ratios of SbSI piezocatalyst mass to the initial MO mass in aqueous solution (●—m_p_/m_MO_ = 200, ▲—m_p_/m_MO_ = 100). Red and blue lines in graph (**b**) represent the best fitted dependence described by Equation (1). Piezocatalysis was performed using Sonic-6 (Polsonic) reactor with ultrasound frequency f = 40 kHz and power P = 480 W.

The piezocatalytic experiments, described above, were accomplished also for other ultrasonic reactors and solutions with various ratios of SbSI piezocatalyst mass to the initial mass of MO (m_p_/m_MO_). As presented in Figure 5, the kinetics of the degradation process depends significantly on the applied amount of piezocatalyst. The values of the reaction rate constant of k_2_ = 0.085(6) s^−1^ and k_3_ = 0.034(3) s^−1^ were determined for m_p_/m_MO_ = 200 and m_p_/m_MO_ = 100, respectively. The decline in the degradation rate and efficiency with the increase in the dye concentration can be attributed to the shielding of the catalyst by the MO dye in solution [35].

**Figure 6:**
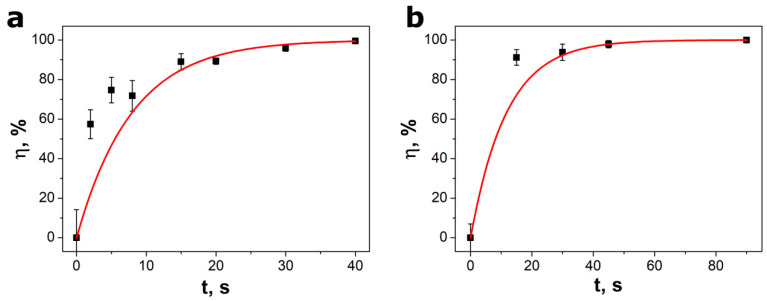
The ratio of piezocatalytic decomposition of methyl orange as a function of ultrasonic irradiation carried out with two different reactors: (**a**) VCX-750 (f = 20 kHz, P = 750 W) and (**b**) Sonic-6 (f = 40 kHz, P = 480 W). Red lines represent the best fitted dependence described by Equation (2).

The efficiency of degradation (η) was calculated using the equation [17,36]:

(2) $$\eta =\frac{C_{0}-C_{t}}{C_{0}}\cdot 100\%=\left(1-e^{-k\cdot t}\right)\cdot 100\%$$

where C_0_ is initial MO concentration and C_t_ means MO concentration after t time of an ultrasonic irradiation. One can see that application of different ultrasonic reactor influences the degradation efficiency (Figure 6). As expected, the decomposition of MO was more intense at higher ultrasonic power (Figure 6a). The piezocatalysis efficiency should also depend on the relation between the frequency of ultrasounds and the resonance frequency of the nanowires [44]. Moreover, a literature in the field of sonochemistry suggests that ultrasonic frequencies can be of paramount importance for some analytical applications [45]. For instance, Doche et al. [46] studied zinc corrosion and oxidation mechanism in ultrasonically stirred electrolytes and proved that cavitation is more powerful at a frequency of 20 kHz than at f = 40 kHz. The piezocatalytic ability of BaTiO_3_ nanoparticles as reference catalysts was tested, too. The degradation efficiency η = 65.1% was achieved after 60 min of ultrasonic irradiation with a Sonic-6 reactor (f = 40 kHz, P = 480 W). It indicates that piezocatalytic activity of BaTiO_3_ nanoparticles is much lower than that in the case of SbSI nanowires.

The reaction rate constant of piezocatalytic degradation of MO by SbSI nanowires was compared with values of this parameter achieved for other piezocatalysts (Table 1). It should be underlined the value of k for SbSI nanowires is over two orders of magnitude larger than rate constant for the most efficient piezocatalysts reported in the literature, i.e., nanowires of La-doped Pb(Zr,Ti)O_3_ (PLZT) [18] and Ba_1−x_Sr_x_TiO_3_ [10].

**Figure 7:**
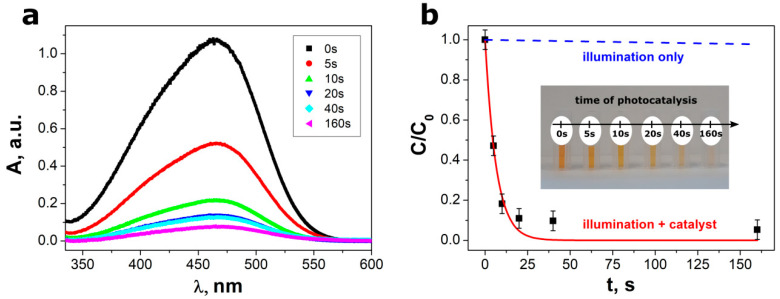
(**a**) An influence of photocatalysis time on UV–vis absorption spectra and (**b**) relative concentrations (C/C_0_) of the MO aqueous solutions. Red line in graph (**b**) represents the best fitted dependence described by Equation (1). Blue dashed line in graph (**b**) shows data registered in control experiments conducted under light illumination without SbSI photocatalyst. The inset in graph (**b**) presents decoloration of MO solution with increase of photocatalysis time.

The additional experiments were performed in order to confirm good catalytic properties of SbSI nanowires. The methyl orange solutions containing SbSI catalyst were subjected to UV light illumination (λ = 365 nm). An influence of photocatalysis time on UV–vis absorption spectra is depicted in Figure 7a. It is clearly visible in Figure 7b, that the presence of SbSI catalyst in MO solution is crucial for dye photodegradation. The light illumination does not have an impact on the concentration of MO dissolved in water without the catalyst. It was proved that 95% of the dye can be degraded in 160 s using a small amount of SbSI nanowires (1 g/L). The relative concentration ratio of methyl orange as a function of illumination time was best fitted with the theoretical dependence described by Equation (1). The large photocatalysis rate constant k_4_ = 9(1) min^−1^ was determined for SbSI nanowires. This value is significantly higher than the rate constant achieved for photocatalytic degradation of methyl orange using SbSI microrods (see Table 2). However, the determined k_4_ parameter is approximately three times lower than value of the reaction rate constant (25.2 min^−1^) reported by Wang et al. [34] for SbSI nanocrystals.

The ultra-high piezocatalytic activity of SbSI nanowires under ultrasonic vibration can be attributed to the several factors. Firstly, SbSI exhibits excellent piezoelectric and electromechanical properties, which have been listed in the introduction section in this paper. Secondly, it is known that the surface of a catalyst acts as the nucleus for cavitation bubbles in the sono-catalytic process [58]. Hence, the large number of nucleus can be formed on the surface of the SbSI nanowires, which possess large surface to volume ratio [37] and their piezoelectric response is very sensitive to external impact. Thirdly, the one-dimensional structure supports the high flexibility and large strain tolerance [8]. The nanowire morphology favors generation of higher piezoelectric potential than that induced in nanoparticles under the same ultrasonic vibration [9]. It was concluded in [35] that, in the case of SbSI microrods, their high photocatalytic activity on the organic pollutants originates from large static dielectric generation in SbSI leading to effective screening the charge carriers. Another important factor was a reduced electron–hole recombination related to a lower dangling bond on the surface of the one-dimensional SbSI rods [35].

**Figure 8:**
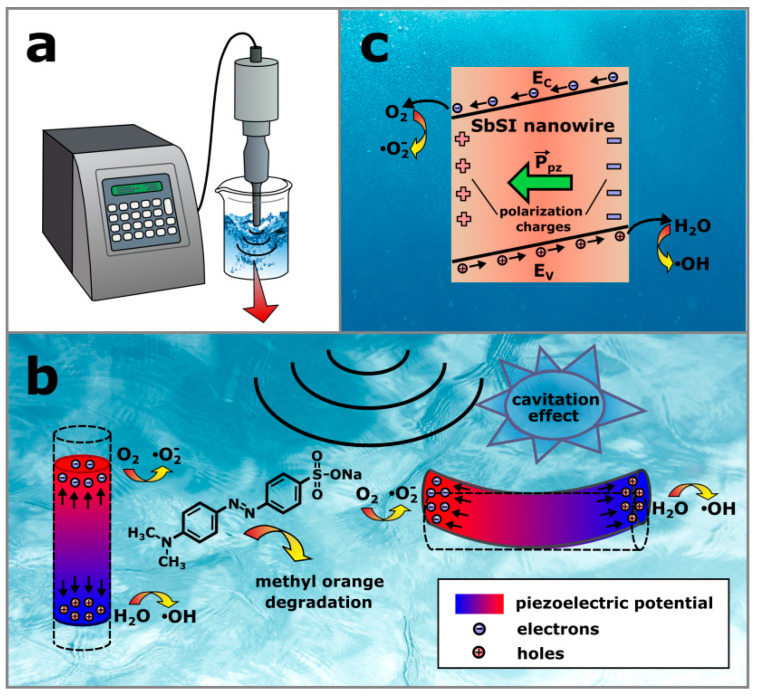
Schematic illustrations of (**a**) an experimental setup, (**b**) piezocatalytic degradation of methyl orange using SbSI nanowires, and (**c**) energy band diagram of SbSI under a piezoelectric potential. Detailed description in the text.

The working principle of the degradation activity through the piezocatalytic effect in SbSI nanowires can be described as follows. When high power ultrasounds are introduced through the sonotrode to the MO–SbSI suspension (Figure 8a), the cycles of compression and rarefaction pressure are created in the liquid medium. The acoustic pressure fluctuates in waveform on the order of 10^5^–10^6^ Pa [9]. Furthermore, microbubbles, filled with a gas, are formed, while a negative pressure is applied to a liquid. After the micrometer-scale bubble reaches its critical size, it collapses. According to Merouani et al. [59], extreme local pressure of 2.5 × 10^8^ Pa is generated within the collapsing bubble. Thus, the nanowires undergo different types of forces, i.e., vertical and lateral, as shown in Figure 8b. They are compressed or bent and a piezoelectric potential is formed as a result of the piezoelectric effect [8]. The conduction (E_C_) and valence bands (E_V_) of SbSI become tilted under a piezoelectric potential (Figure 8c). The piezoelectric polarization (P_pz_), induced by ultrasonic vibration, provides driving force for the separation of intrinsic electrons (e^−^) and holes (h^+^) in the SbSI nanowires. Therefore, these charge carriers move in opposite directions towards the positive and negative surfaces, where they react with the adsorbed molecules to produce various free radicals [47]. The oxygen molecules, present in the gas bubbles in the liquid, are adsorbed on the SbSI surface [33]. Then, they receive electrons from the conduction band and form the free radicals on the positive surface [8,10,20,47]:

(3) $$O_{2}+e^{-}\to {}^{•}O_{2}^{-}$$

The water molecules are randomly distributed in the methyl orange solution. They can act as acceptors or donors due to different electric charges at the hydrogen and oxygen ends. The surface of the SbSI semiconductor with the positive carriers (holes) attracts the negative end of the polar molecule of water and adsorbs it [40]. As a result, H^+^ ions and hydroxyl radicals (•OH) are created on the negative surface [8,10,15,47]

(4) $$H_{2}O+h^{+}\to H^{+}+{}^{•}OH$$

In the next stage, H^+^ ions are adsorbed on the semiconductor surface with the negative carriers (electrons). Then, hydrogen peroxide is formed as a product of reaction between H^+^ ions and •O_2_^−^ radicals [15]:

(5) $$2H^{+}+{}^{•}O_{2}^{-}+e^{-}\to H_{2}O_{2}$$

H_2_O_2_ molecule diffuses to be absorbed on the negatively charged surface of SbSI nanowire and reacts with H^+^ ions and free electrons:

(6) $$H_{2}O_{2}+H^{+}+e^{-}\to H_{2}O+{}^{•}OH$$

Finally, the generated highly reactive radicals react with the adsorbed MO molecule leading to its degradation [8,10,14,20]. It should be noted that •O_2_^−^ [35,36] and •OH radicals [36] were found as the primary reactive species in the organic pollutants degradation carried out with SbSI as a photocatalyst.

The catalytic degradation of methyl orange is accompanied by three possible chemical processes: demethylation, methylation, and hydroxylation [47,54,60]. Demethylation leads to substitution of the methyl group with a hydrogen atom. When hydroxylation takes place, the benzene ring in the original MO molecule is replaced with a hydroxyl group. Demethylation is more favorable than hydroxylation, which results in the formation of largest number of intermediates [60]. Usually, piezocatalytic decomposition of methyl orange involves production of byproducts with mass-to-charge ratios of 290 [10,47], 304 [10], 306 [47], and 320 [10,47]. They can be further split into smaller intermediates, which are finally mineralized into inorganic products, i.e., CO_2_ [10,54], H_2_O [10,54], NO_3_^−^ [54,61], SO_4_^2−^ [61], and NH_4_^+^ [61]. However, the recognition of the exact mechanism of MO degradation using SbSI nanowires and formation of intermediates needs additional experiments. This will be investigated in the future.

The method of water purification, proposed in this paper, is a relatively cheap. The cost of SbSI catalyst production was evaluated taking into account the electrical energy consumption and the expenses spent on purchasing the high purity reagents, i.e., antimony, sulfur, iodine, and ethyl alcohol. The cost of SbSI nanowires fabrication amounts to 2.11 USD (1.8 EUR) on average per 1 g of this nanomaterial. For example, this is approximately 2.2 times lower than value of BaTiO_3_ nanoparticles that can be purchased commercially from Sigma-Aldrich (Merck). It should be underlined that energy requirements for piezo- and photocatalysis using SbSI nanowires are negligible due to the extremely short time (40 s) needed to achieve high efficiency of organic dye removal (99.5%).

## 4. Conclusions

The ferroelectric SbSI nanowires, prepared sonochemically, have exhibited ultrahigh degradation activity to decompose the methyl orange dye under ultrasonic vibration or under light illumination. As expected, the decomposition of MO was more intense when higher ultrasonic power was applied. The increase in the ratio of piezocatalyst mass to the initial dye concentration led to enhancement of the degradation rate. The reaction kinetics has been found to follow a pseudo-first-order law. Extremely high degradation efficiency of 99.5% was achieved for SbSI nanowires after their ultrasonic irradiation for only 40 s.

The determined reaction rate constant of 0.126(8) s^−1^ was over two orders of magnitude larger than this parameter reported in the literature for the most efficient piezocatalysts. However, this value is slightly lower than the reaction rate constant of photocatalytic degradation of methyl orange achieved for the same nanomaterial or SbSI nanocrystals fabricated via the ball-milling method. The ultrahigh catalytic activity of SbSI nanowires under ultrasonic vibration may result from their excellent piezoelectric and electromechanical properties, large surface-to-volume ratio, as well as their one-dimensional structure that supports high flexibility and strain tolerance.

The piezo/photocatalytic decomposition of organic dye using SbSI nanowires, presented in this paper, is simple and inexpensive, which is crucial for industrial scale applications. Remarkable piezo/photocatalytic properties of SbSI nanowires are promising for their future utilization in environmental remediation and in the field of renewable carbonfree energy production from water splitting.

## Figures and Tables

**Table 1:** A comparison of efficiencies of different piezocatalysts used for ultrasound-assisted degradation of methyl orange (used abbreviations: BCTZ—(Ba_0.85_Ca_0.15_)(Ti_0.9_Zr_0.1_)O_3_; f—ultrasounds frequency; k—reaction kinetics rate constant; m_p_/m_MO_—the ratio of piezocatalyst mass to the initial mass of methyl orange in aqueous solution; NFs—nanofibers NPs—nanoparticles; NWs—nanowires; PLZT—La-doped Pb(Zr,Ti)O_3_; PZT—Pb(Zr,Ti)O_3_; P/P_d_—ultrasounds power or power density; t—ultrasonic vibration time; η—degradation efficiency).

Piezocatalyst	m_p_/m_MO_	f, kHz	P or P_d_	t	η, %	k, min^−1^	Year	Reference
BaTiO_3_ NWs	200	40	120 W	160 min	95	0.0164	2019	[8]
BaTiO_3_ NWs	200	40	80 W	160 min	~90	~0.017	2018	[9]
BaTiO_3_ NWs	200	40	0.1 W/cm^2^	120 min	79	0.0132	2018	[10]
BaTiO_3_ NPs	200	40	80 W	-	-	0.019	2018	[11]
BaTiO_3_ NPs	200	40	480 W	60 min	65.1	-	2020	this paper
BaTiO_3_/Ag NPs	200	40	120 W	120 min	81	0.0162	2018	[47]
Ba_1−x_Sr_x_TiO_3_ NWs	200	40	0.1 W/cm^2^	120 min	100	0.0196	2018	[10]
BCTZ NWs	200	40	120 W	150 min	65	0.0071	2018	[48]
PLZT NWs	200	40	120 W	160 min	97	0.02	2019	[18]
PZT NWs	-	40	120 W	-	-	0.0155	2019	[18]
Na_0.5_K_0.5_NbO_3_	800	40	-	100 min	77	-	2020	[16]
NiO NPs	100	37	160 W	60 min	96	-	2019	[17]
ZnO@TiO_2_ NFs	100	40	-	120 min	60	-	2017	[20]
SbSI NWs	200	20	750 W	40 s	99.5	7.6(5)	2020	this paper
	200	40	480 W	45 s	97.9	5.1(4)		
	200	40	480 W	45 s	84.0	2.0(2)		

**Table 2:** The values of reaction kinetics rate constants for photocatalytic decomposition of methyl orange using various materials (used abbreviations: m_ph_/m_MO_—the ratio of photocatalyst mass to the initial mass of methyl orange in aqueous solution; MRs—micro-rods; NCs—nanocrystals; NPs—nanoparticles; NRs—nanorods; NSs—nanosheets; NWs—nanowires; t_ph_—time of photocatalysis; η—degradation efficiency).

Photocatalyst	m_ph_/m_MO_	Illumination	t_ph,_ s	η, %	k, min^−1^	Year	Reference
Ag_3_PO_4_	250	xenon lamp (300 W)	240	98	-	2011	[49]
Ag_2_O/Ag_2_CO_3_	25	halogen lamp (150 W)	300	100	0.92	2013	[50]
AgBr/graphene	240	xenon lamp (500 W)	480	100	0.72	2015	[51]
CdS/C	50	xenon lamp (500 W)	2400	97	-	2013	[52]
TiO_2_ NSs	71.4	xenon lamp (300 W)	2400	84	-	2010	[53]
TiO_2_ NPs	20	sunlight irradiation	7200	98	-	2016	[54]
ZnO NRs	-	mercury lamp (125 W)	4800	100	-	2011	[55]
WS_2_ NSs	50	mercury lamp (300 W)	6000	96	-	2015	[56]
Sb_2_S_3_	25	halogen lamp (500 W)	1800	97	-	2008	[57]
SbSI NCs	33.3	xenon lamp (400 mW/cm^2^)	10	99	25.2	2018	[34]
SbSI MRs	50	solar simulator (1.5G AM)	1200	97	0.19	2016	[35]
SbSI NWs	33.3	UV lamp (300 W)	160	95	9(1)	2020	this paper
